# Mixed-Potential Gas Sensors Using an Electrolyte Consisting of Zinc Phosphate Glass and Benzimidazole

**DOI:** 10.3390/s17010097

**Published:** 2017-01-05

**Authors:** Takafumi Akamatsu, Toshio Itoh, Woosuck Shin

**Affiliations:** National Institute of Advanced Industrial Science and Technology (AIST), Inorganic Functional Materials Research Institute, 2266-98, Anagahora, Shimo-Shidami, Moriyama-ku, Nagoya-shi 463-8560, Japan; itoh-toshio@aist.go.jp (T.I.); w.shin@aist.go.jp (W.S.)

**Keywords:** gas sensor, mixed-potential, zinc phosphate glass, hydrogen

## Abstract

Mixed-potential gas sensors with a proton conductor consisting of zinc metaphosphate glass and benzimidazole were fabricated for the detection of hydrogen produced by intestinal bacteria in dry and humid air. The gas sensor consisting of an alumina substrate with platinum and gold electrodes showed good response to different hydrogen concentrations from 250 parts per million (ppm) to 25,000 ppm in dry and humid air at 100–130 °C. The sensor response varied linearly with the hydrogen and carbon monoxide concentrations due to mass transport limitations. The sensor responses to hydrogen gas (e.g., −0.613 mV to 1000 ppm H_2_) was higher than those to carbon monoxide gas (e.g., −0.128 mV to 1000 ppm CO) at 120 °C under atmosphere with the same level of humidity as expired air.

## 1. Introduction

Expired air contains over 100 different gases in a humid atmosphere at low concentrations, ranging from sub parts per billion (ppb) to parts per million (ppm), including hydrogen (H_2_), carbon monoxide (CO), and various volatile organic compounds (VOCs) [1,2,3,4,5,6]. Expired air can provide information that is useful for monitoring human health. The concentration of H_2_ in expired air has been used to monitor microbial metabolism in the colon [1,2,3,4], since H_2_ is produced by intestinal bacteria. High-performance gas sensors provide a simple and non-invasive method to monitor expired air, and are therefore one of the most promising approaches for the early detection of diseases.

Mixed-potential gas sensors using yttria-stabilized zirconia (YSZ) electrolyte have been investigated for the detection of H_2_, CO, nitric oxide, ethanol, and methane [7,8,9,10,11]. These gas sensors must be operated at temperatures above 500 °C to provide both satisfactory response rates against target gases and good gas selectivity. These gas sensors require a heater to maintain the operating temperature above 500 °C, which results in high power consumption. Mixed-potential gas sensors that use electrolytes with high proton conductivity—such as Nafion, zirconium phosphate, and antimonic acid—have been reported to function at reduced operating temperatures [12,13,14,15]. However, these electrolytes must be impregnated with water to achieve high proton conductivity, which necessitates the inclusion of a complicated humidity control system to allow these sensors to operate at room temperature and under high humidity. Moreover, these gas sensors show low selectivity for H_2_ against CO. One approach to overcoming this challenge is to use anhydrous proton conductors at an intermediate temperature between 100 and 300 °C. However, to the best of our knowledge, no mixed-potential gas sensors using a high-proton-conductivity electrolyte at intermediate temperatures have been reported (Table 1).

Oine et al. and Kato et al. have successfully prepared an anhydrous electrolyte that demonstrates high proton conductivity at temperatures between 100 and 200 °C by the reaction of zinc metaphosphate glass with benzimidazole [16,17]. This material has been investigated as an electrolyte in electrochemical devices that operate at intermediate temperatures, such as fuel cells. Thus, we suggest that this anhydrous electrolyte has the potential to be applied as a mixed-potential gas sensor.

In this study, we prepared a mixed-potential gas sensor using an electrolyte consisting of zinc metaphosphate glass and benzimidazole, and the sensor’s response to H_2_ gas in both humid and dry air was investigated. An initial investigation of the sensor’s response to CO in humid air was also conducted to determine its selectivity for H_2_ against other gases.

## 2. Materials and Methods

A glass with a nominal molar ratio of 1:1 ZnO:P_2_O_5_ was prepared by melting a batch mixture of the commercially available reagent-grade chemicals ZnO and H_3_PO_4_ in a platinum crucible at 1100 °C in air for 30 min. The melt was poured onto an iron plate and quenched by iron pressing to make glass flakes. The glass flakes were milled using an alumina mortar and pestle to below 100 μm in diameter. The glass powder was mixed with benzimidazole, and the mixture was heated at 170 °C for 12 h to make an electrolyte. The weight ratio of the glass powder and benzimidazole was 1:3. Details of the procedures and electrolyte properties have been previously reported [17].

**Figure 1:**
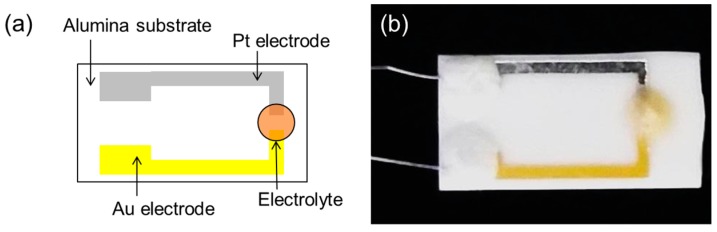
The structure of the sensor: (**a**) schematic illustration; and (**b**) optical image.

The sensor element we constructed is shown schematically and as a photograph in Figure 1. The electrolyte was deposited on an alumina substrate with platinum (Pt) and gold (Au) electrodes, with both the electrode gap and electrode width being 1 mm. Finally, the electrolyte-coated substrate was heated from behind by placing the assembly on a hotplate, resulting in the formation of an electrolyte membrane on the substrate.

The sensor element was placed in a test chamber and heated to 80–130 °C in an electrical tube furnace. Synthetic dry air was introduced into the chamber for 20 min, and a gas mixture of H_2_ or CO in synthetic dry air was then injected for 20 min. The gas mixture flow rate was 200 mL/min. Synthetic dry air and H_2_ or CO in synthetic air were prepared by mixing 99.99% N_2_ gas, N_2_-balanced 5% H_2_ gas, N_2_-balanced 5% CO gas, and 99.5% O_2_ gas. The O_2_ content of synthetic dry air and that of H_2_ or CO in synthetic dry air were both 20 vol %. The H_2_ or CO gas concentrations in synthetic dry air were controlled at values of 250, 500, 1000, 2500, 5000, 10,000, and 25,000 ppm. Synthetic humid air was prepared by passing air through ion-exchanged water at 25 °C before flowing to a test chamber.

The potential difference (electromotive force, EMF) between Pt and Au electrodes of the sensor element was recorded using a data logger (midi Logger GL220; Graphtec Corp., Yokohama, Japan). During the potential difference measurements, the Pt electrode was always connected to the positive terminal of the data logger. The EMFs of the sensor element in the air and gas mixtures are denoted as *V_a_* and *V_g_*, respectively. The sensor response (*ΔV*) is defined as *ΔV* = *V_a_* − *V_g_*. 

## 3. Results and Discussion

**Figure 2:**
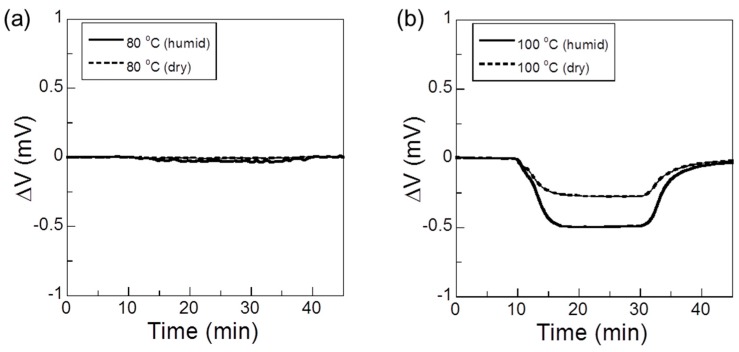

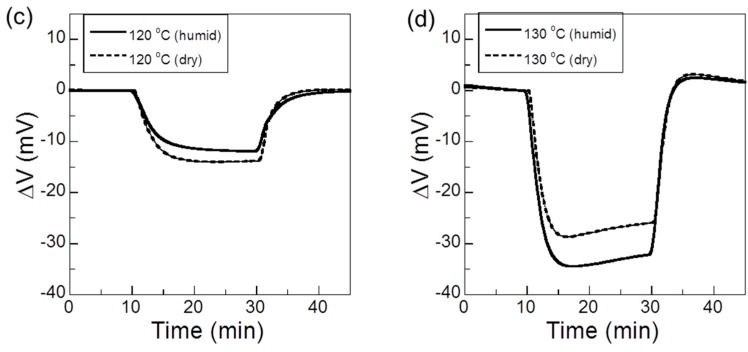
Sensor responses to 25,000 ppm H_2_ in humid and dry air at (**a**) 80 °C; (**b**) 100 °C; (**c**) 120 °C; and (**d**) 130 °C.

Figure 2 shows that upon exposure to 25,000 ppm H_2_ in humid and dry air at 80 °C, 100 °C, 120 °C, and 130 °C, the sensor’s response increased with increasing operating temperature. Miura et al. described the potential response upon exposure to hydrogen in humid gas of a solid-state sensor that used a proton-conductor [12]. The EMF depends on the electrochemical oxidation and reduction reactions occurring at the Pt and Au electrodes:

(1) H_2_ → 2H^+^ + 2e^−^

(2) (1/2)O_2_ + 2H^+^ + 2e^−^ → H_2_O

A mixed potential is established when the electron transfer rates of the hydrogen oxidation and the oxygen reduction are same. Since the kinetics of the sensor’s Pt and Au electrodes are different from those of the oxidation of H_2_ to water, the mixed-potential generated at each electrode varies, and thus *ΔV* varies in accordance with the H_2_ gas concentration. We reported that the sensor response to H_2_ in synthetic dry air at 150 °C is higher than that at 130 °C [18]. However, in this paper, when the sensor response to H_2_ was investigated at 140 °C, the EMF was unstable and gradually decreased over the measuring time, and the sensor showed no response to H_2_ gas (not pictured). This behavior may result from changes in the electrochemical properties of the electrolyte due to volatilization of the benzimidazole in the electrolyte, because the flash point of benzimidazole was 143 °C [19]. Therefore, the optimum operating temperature of the sensor is below 140 °C. In general, the sensor responses are similar at each temperature. At 120 °C, the sensor response in dry air is higher, whereas for the other temperatures, the sensor response in humid air is higher. The electrolyte derived from zinc metaphosphate glass and benzimidazole is an anhydrous proton-conducting material, and has good water durability [16,17]. Therefore, the electrical properties of the electrolyte were not changed by water vapor, and the sensor responses using the electrolyte was considered to be seen even in humid air. However, the difference between the sensor responses to H_2_ in dry air and humid air was significant. In future study, we will investigate the cycle characteristics and error evaluation of the sensor response. 

**Figure 3:**
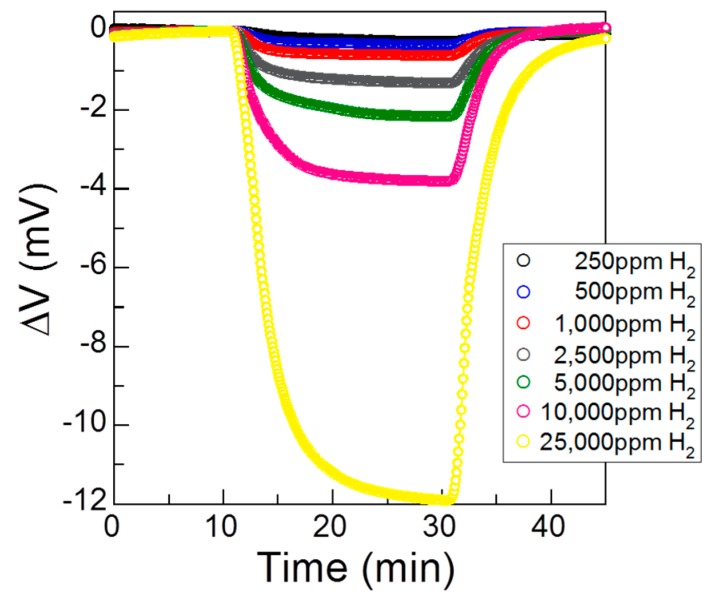
Sensor response to different concentrations of H_2_ in humid air at 120 °C.

**Figure 4:**
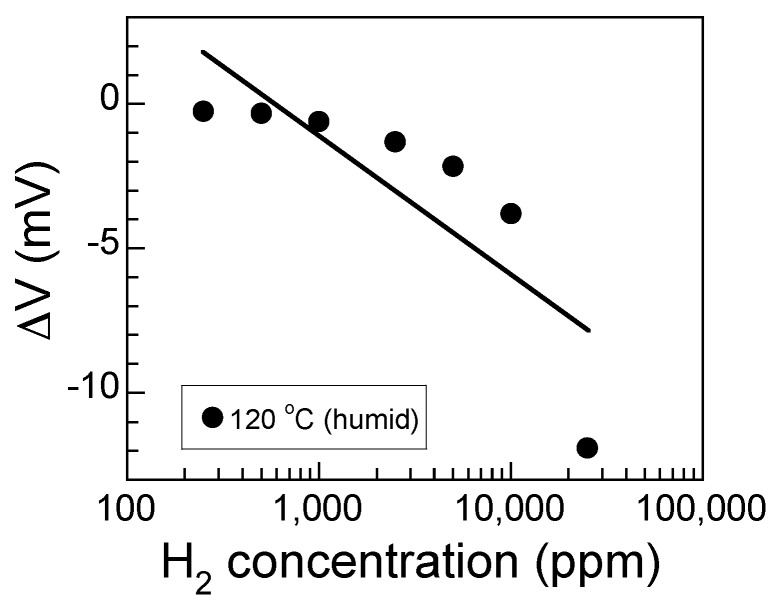
The relationship between sensor response and the log of the H_2_ concentration in humid air at 120 °C. The solid line shows the least-squares linear fit.

Figure 3 shows the sensor responses to H_2_ concentrations ranging from 250 ppm to 25,000 ppm in humid air at 120 °C. These results show that the *ΔV* of the sensor began to decrease rapidly on exposure to H_2_, and the response was stronger for higher concentrations of H_2_. Figure 2 shows that the difference between the sensor responses to H_2_ in dry air and humid air was large at this stage. In order to detect H_2_ with high accuracy, error evaluation of the sensor response under different humidity conditions must be investigated. The model designed by Miura et al. suggests that a linear relationship exists between the logarithm of the H_2_ concentration and *ΔV*, assuming a Tafel-like equation for the polarization behavior of the oxidation and reduction reactions at the electrode [12]. The *ΔV* can thus be expressed in the form:

(3) *ΔV* = *a* + *b* log *C_H_*_2_

where *a* and *b* are constants, and *C_H_*_2_ is the concentration of H_2_. Figure 4 shows the relationship between the sensor response and the logarithm of the H_2_ concentration. The least squares regression analysis of the sensor can be expressed as follows:

(4) *ΔV* = 13.1 − 4.7 log *C_H_*_2_, *R* = 0.819

where the *C_H_*_2_ is the concentration of H_2_, and *R* is the correlation coefficient. The sensitivity of the sensor was −4.7 mV/decade. However, no good linear relationship between the logarithm of H_2_ concentration and *ΔV* of the sensor was obviously found. 

According to Garzon et al., if the concentration of the sensed gas (H_2_) is much lower than the concentration of O_2_, a linear relationship between the H_2_ concentration and *ΔV* exists because of the diffusional mass transport limitation [8]. In this case, *ΔV* can be expressed as

**Figure 5:**
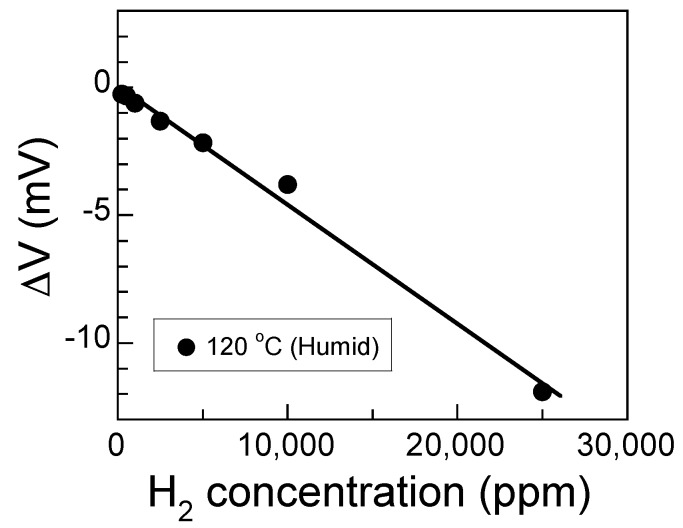
The relationship between the sensor response and the H_2_ concentration in humid air at 120 °C. The solid line shows the least-squares linear fit.

(5) *ΔV* = *a* + *K C_H_*_2_

where *a* and *K* are constants. Figure 5 shows the relationship between sensor response and the H_2_ concentration. The sensor response was adequately linear. The least squares regression analysis of the sensor can be expressed as follows:

(6) *ΔV* = 0.021 − 0.00046 *C_H_*_2_, *R* = 0.996

where *C_H_*_2_ is the concentration of H_2_, and *R* is the correlation coefficient. The sensitivity of the sensor was −0.00046 mV/ppm. *R* for Equation (6) was higher than that for Equation (4). This suggested that the oxidation reaction of H_2_ may be mass transport-limited. 

**Figure 6:**
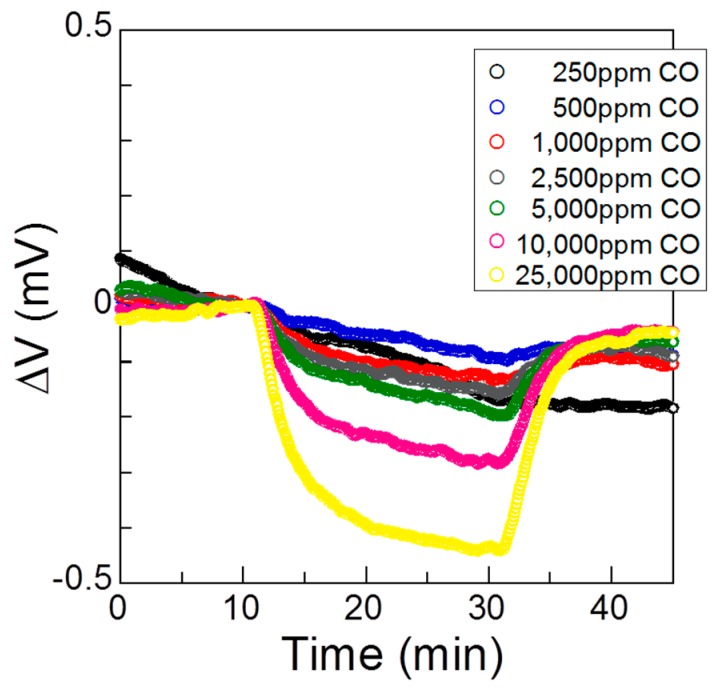
Sensor responses to different concentrations of CO in humid air at 120 °C.

Figure 6 shows the sensor’s responses to CO in humid air at 120 °C and at concentrations ranging from 250 to 25,000 ppm. When the sensor was exposed to these conditions, its *ΔV* decreased rapidly, and the response decreased as the CO concentration increased. In CO gas sensing, the oxidation reaction of CO can take place simultaneously at both the Pt and Au electrodes:

(7) CO + H_2_O → CO_2_ + 2H^+^ + 2e^−^

**Figure 7:**
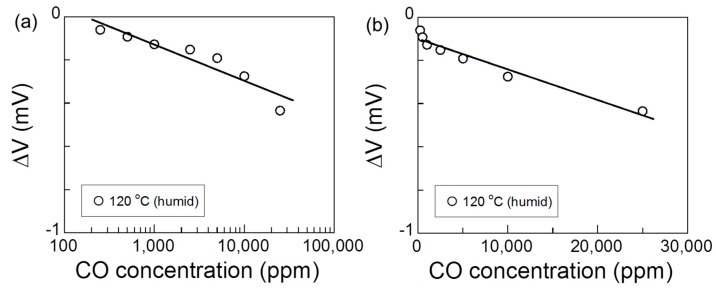
(**a**) The relationship between the sensor response and the logarithm of the CO concentration and (**b**) the relationship between the sensor response and the CO concentration in humid air at 120 °C. The solid lines show the least-squares linear fit.

Figure 7 shows the relationship between the sensor response and the logarithm of the CO concentration, and the relationship between the sensor response and the CO concentration in humid air at 120 °C. The equations of the relationship between the sensor response and CO concentration were determined from Equations (3) and (5), respectively:

(8) *ΔV* = 0.376 − 0.168 log *C_CO_*, *R* = 0.939

(9) *ΔV* = 0.101 − 1.015 × 10^−6^*C_CO_*, *R* = 0.977

where *C_CO_* is the concentration of CO and *R* is the correlation coefficient. Since *R* is slightly larger for Equation (9) than for Equation (8), the oxidation reaction of CO may be mass-transport-limited, as seen for the H_2_ gas sensing. 

The sensor responses to 1000 ppm H_2_ and 1000 ppm CO were determined to be −0.613 mV and −0.128 mV, respectively, in humid air at 120 °C. These responses are lower than those seen for mixed-potential gas sensors that used proton conductor operating at around room temperature (−190 mV for 1000 ppm H_2_ and −120 mV for 1000 ppm CO) [12]. The value of H_2_ selectivity against CO (*S*_*H*2*/CO*_) was determined using the equation *S*_*H*2*/CO*_ = *ΔV*_*H*2_/*ΔV_CO_*, where *ΔV*_*H*2_ is the sensor’s response at 1000 ppm H_2_, and *ΔV_CO_* is the sensor response at 1000 ppm CO. The *S*_*H*2*/CO*_ of our sensor was 4.8, and that of the sensor using a proton conductor operating at around room temperature was 1.6 [12]. 

Semiconductor gas sensors using n-type SnO_2_ have been investigated for several decades for their high response to inflammable gases, such as H_2_, CO, CH_4_, and C_2_H_5_OH [20,21]. However, their low selectivity for H_2_ over CO was regarded as a problem to be solved. Moon et al. focused on the use of a CuO-doped SnO_2_-ZnO composite material to improve the gas selectivity, and reported a selectivity of ~4 for H_2_ (200 ppm) over CO (200 ppm) at 350 °C [21]. The sensor reported herein—which used an electrolyte consisting of zinc metaphosphate glass and benzimidazole—showed a higher selectivity for H_2_ against CO than the sensor reported by Moon et al. However, our sensor’s response to H_2_ is currently inferior, thus optimization of the sensor design is required to achieve a greater response. Further investigations of the sensor’s response to other interfering species (such as CH_4_ and VOCs) will be conducted in the future.

## 4. Conclusions

In this study, we have investigated the responses of a mixed-potential gas sensor that used a proton conductor composed of zinc metaphosphate glass and benzimidazole for the detection of H_2_ (produced by intestinal bacteria) and CO gas (an interference in expired air). The gas sensor showed good sensor response to H_2_ in the concentration range 250–25,000 ppm under atmosphere having the same level of humidity as expired air at 120 °C. The mixed-potential gas sensor without a heater can detect gases under the atmosphere at temperatures in the range 100–130 °C. The sensor response varied linearly with the hydrogen and carbon monoxide concentrations, as expected under mass-transport-limited conditions. The gas sensor showed high gas selectivity for H_2_ against CO (*S*_*H*2*/CO*_ = 4.8).

## Figures and Tables

**Table 1:** Typical examples of mixed-potential gas sensors for H_2_ detection. YSZ: yttria-stabilized zirconia.

Sensor Structure			Sensing Temperature	H_2_ Concentration	References
Sensor Materials	Sensing Electrode	Reference Electrode			
YSZ	ZnO	Pt	400–600 °C	50–500 ppm	[7]
Sb_2_O_5_∙4H_2_O	Pt	Au	Room temperature	200–10,000 ppm	[12]
Zr(HPO_4_)_2_∙nH_2_O	Pt	Ag	Room temperature	300–10,000 ppm	[14]
Sn_0.9_In_0.1_P_2_O_7_	Pt/C	Pt	30 °C	100–30,000 ppm	[15]
